# Climate change impacts on fish in the Baltic Sea

**DOI:** 10.1111/jfb.70582

**Published:** 2026-07-26

**Authors:** Örjan Östman, Meri Kallasvuo, Sanna Kuningas, Antti Lappalainen, Noora Mustamäki, Rahmat Naddafi, Lauri Saks, Jens Olsson

**Affiliations:** ^1^ Department of Aquatic Resources Swedish University of Agricultural Sciences Uppsala Sweden; ^2^ Natural Resources Institute Finland (Luke) Helsinki Finland; ^3^ Estonian Marine Institute University of Tartu Tartu Estonia; ^4^ Institute of Veterinary Medicine and Animal Sciences Estonian University of Life Sciences Tartu Estonia

**Keywords:** adaptations, Baltic Sea, community, global change, management, marginal seas, non‐indigenous species

## Abstract

The Baltic Sea is a marginal sea with strong abiotic gradients harbouring fish of both marine and freshwater origin. Here we review the empirical evidence for how changes in climate variables have impacted different fish and fish communities in the Baltic Sea. Studies on responses to temperature, salinity and hypoxia dominate the literature, with most evidence for negative influences on reproduction and feeding areas for demersal fish of marine origin. In contrast, warmer waters benefit the reproduction and growth of some smaller pelagic and many coastal fish species of freshwater origin, but responses on population or community levels are elusive. Several responses to climate change have further been mediated through changes in prey abundance and quality. Studies investigating adaptations to climate change indicate that fish are adapting, partly counteracting climate impact. A precautionary approach to management is therefore important to safeguard climate change adaptation and reduce deleterious impacts as the impact on the Baltic Sea from a changing climate likely will be even more pronounced in the coming decades.

## 1 INTRODUCTION

Unprecedented rates of climate change will influence a range of physiochemical conditions across all seas, most notably manifested as increases in water temperature (Abraham et al., 2013; Belkin, 2009), reduced ice cover (Sadatzki et al., 2020), hypoxia (Limburg & Casini, 2018), ocean acidification (Doney et al., 2020), stronger stratification (Liblik & Lips, 2019) and raised sea levels (Meier et al., 2022; Nerem et al., 2018). Although the net effects may differ between seas, climate change will also impact salinity (Meier et al., 2022), solar radiation (Meier et al., 2022) and the run‐off of nutrients, organic matter and sediments from terrestrial habitats (Viitasalo & Bonsdorff, 2022). All these physiochemical changes will in turn affect the ecology and evolutionary processes in marine ecosystems.

Marginal seas, estuaries and coastal areas have so far warmed at comparably faster rates than the larger oceans (Belkin, 2009). These environments typically have strong abiotic gradients (Meier et al., 2022), hence resulting in higher spatial turnover of populations and species (Johannesson & Andre, 2006; Östman, Olsson, et al., 2017). In addition, marginal seas are often more directly affected by terrestrial processes through run‐off (Viitasalo & Bonsdorff, 2022) and are among the most human‐impacted marine areas (Blenckner et al., 2021; Halpern et al., 2008). Thus, biotic responses to climate change in marginal seas may be more diverse and stronger than in oceans in general.

The Baltic Sea is a brackish marginal sea in the northern hemisphere with evident gradients in temperature, salinity, nutrient concentrations and water colour (Meier et al., 2022; Ojaveer & Kalejs, 2005; Saraiva et al., 2019). It is under substantial anthropogenic impact from eutrophication, fishing, pollution, habitat exploitation and non‐indigenous species (Blenckner et al., 2021; Reckermann et al., 2022). Surface water temperature has increased on average by 2°C since 1990 (Siegel & Gerth, 2019), whereas salinity levels have been more variable (Ojaveer & Kalejs, 2005) due to variation of saltwater influxes from the North Sea and freshwater run‐off from rivers. The latter is expected to increase, especially in the northern part where precipitation is predicted to increase by 30% (Andersson et al., 2015) potentially leading to decreasing salinity in a longer time perspective, but decadal variation is unpredictable (Saraiva et al., 2019). The warmer water in combination with historic high nutrient loadings has resulted in hypoxia in deeper parts of the Baltic Sea, predicted to expand further with increasing warmer temperatures (Saraiva et al., 2019). The warmer water temperatures have also resulted in reduced ice coverage and shorter periods with ice (Dutheil et al., 2022; Meier et al., 2022).

In the Baltic Sea, the natural abiotic gradients result in marked spatial differences in the structure and composition of fish communities. In southern and offshore areas of the Baltic Sea, species of a marine origin dominate (Hiddink & Coleby, 2012; Pecuchet et al., 2016, 2020). These species tend to have a population structure with few but large subpopulations/stocks of individuals moving larger distances (Johannesson & Andre, 2006; Östman, Olsson, et al., 2017). Fish of freshwater origin dominate in sheltered coastal and northern areas of the Baltic Sea (Olsson et al., 2012; Pekcan‐Hekim et al., 2016). These species often constitute many smaller subpopulations with shorter migration distances (Östman, Olsson, et al., 2017), except for anadromous fish. Hence, the impacts of climate change on Baltic Sea fish communities are likely area‐ and species‐specific.

Here we review and synthesise the empirical evidence on responses of fish in the Baltic Sea to changes in climate variables. Our primary question is what are the observed responses and implications of climate‐related variability on individual fish (e.g., fecundity, body growth, mortality, movements), fish populations (e.g., abundance, distributions) and fish communities (diversity, composition) in the Baltic Sea? We therefore divide the response of climate variables at three hierarchical levels: Individuals, Populations and Communities. Secondly, we address how changes in climate variables influences fish communities with different habitat requirements and evolutionary origins, more specifically (i) demersal‐ and (ii) pelagic species of marine origin, (iii) coastal and (iv) migratory (anadromous) fish and (v) non‐indigenous species, including new visiting or fugitive species? Finally, we address what are the evidence for adaptive plastic and evolutionary responses in fish in the Baltic Sea and how adaptive responses may impact individual and population performance?

## 2 METHOD

We applied the ‘Population‐Exposure‐Outcome’ framework to identify relevant literature. It is considered suitable for qualitative reviews on risk assessment and causes of phenomena (Munn et al., 2018). The number of studies identified and excluded at each step is shown in Figure 1.

**FIGURE 1:**
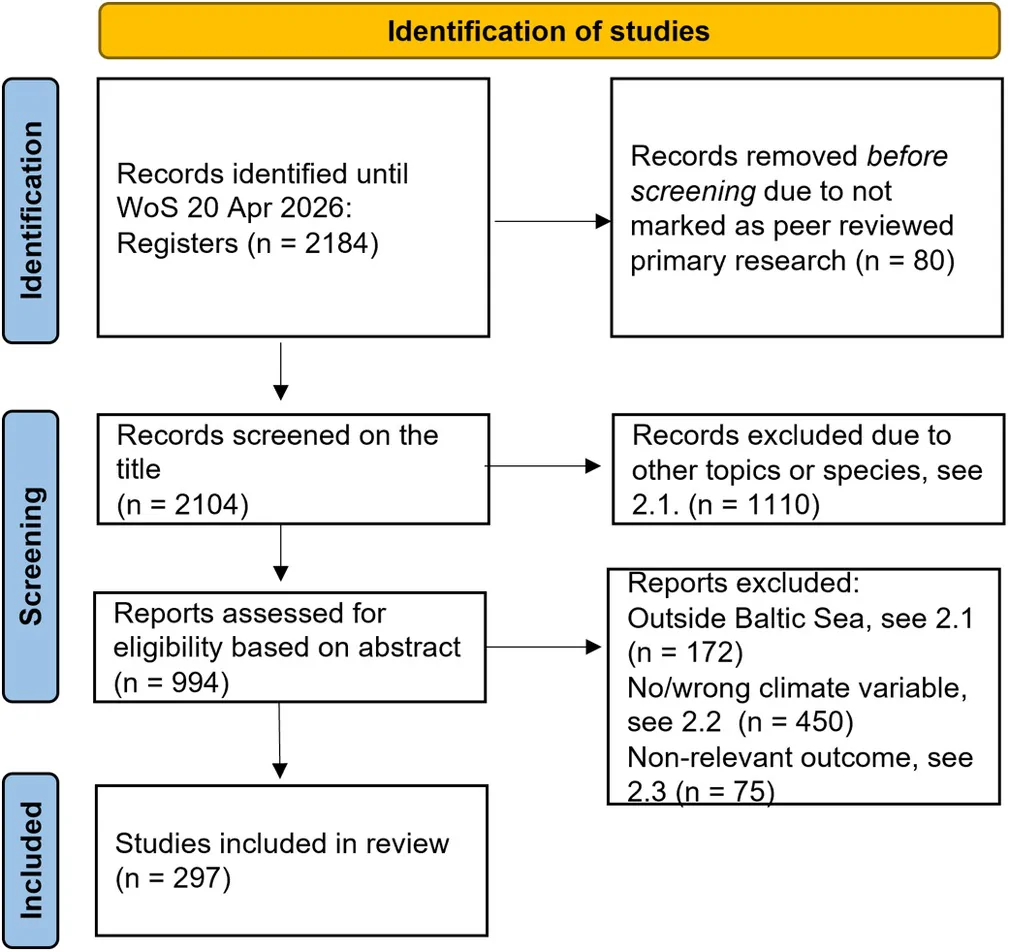
Overview of the number of studies included and excluded at the different steps in the selection process of literature.

### 2.1 Population – Area and species studied

We searched for studies on all vertebrate fish in the brackish Baltic Sea, ICES subdivisions 22–32, from the Öresound (SD22) and Danish straits (SD23) to the Gulf of Bothnia (SD30–31) and Gulf of Finland (SD32) (Figure S1). We did not include Kattegat (SD21) because the fish fauna here is more similar to the North Sea (Johannesson & Andre, 2006). We also excluded studies from freshwater systems.

### 2.2 Exposure – Climate variables

This review includes studies of fish responses to changes in the abiotic climate variables listed by Helcom (2021): temperature, atmospheric circulation, ice, solar radiation, salinity, stratification and oxygen levels, precipitation, terrestrial run‐off, acidification, nutrient loading, sea level, wind, wave and sediment transportation. Of these variables, we omitted atmospheric circulation, solar radiation, sea level, precipitation and sediment transportation as we considered them to have low direct impact on fish. Some of these variables may have evident indirect effects on fish in the Baltic Sea via impacts on salinity, temperature, or oxygen levels, and other factors (e.g., both atmospheric circulation and precipitation drive variation in salinity), but for consistency, we settled for a focus only on the proximate climate drivers impacting fish.

### 2.3 Outcome – Fish responses

We consider responses to climate variables at three hierarchical levels; (i) individual traits and performance, (ii) species (including populations or stocks) and (iii) community level. For the individual level, we restrict studies to physiological responses, food intake, predation and condition, life‐history traits (phenology, fecundity, body growth, maturation, mortality) and movement/migration. We do hence not include studies on otolith chemistry or stable isotopes. At the population level, we consider abundance, recruitment, spatial distribution and size structures, but not spatial population structures or genetic diversity. At the community level, we considered species composition (relative abundance), diversity and trophic structure of fish communities, but not studies where fish were integrated with other taxonomic groups.

### 2.4 Literature search

We used Web of Science (WoS) to search its Core Collection database for studies published until 20 April 2026 with no restriction on publication year, and in ‘All fields’ using the search string:

(“Baltic Sea” OR “Bothni*”) AND (“fish*” OR “cod” OR “herring”)

AND

(“climate” OR “temperature” OR “salin*” OR “oxygen” OR “hypoxi*” OR “ice cover” OR “stratification” OR “acid*” OR “pH” OR “run‐off” OR “wave”),

Where the first part corresponded to ‘Population’ and the second part to ‘Exposure’. We did not use any search string related to ‘Outcome’ because we here consider a wide range of response variables and instead removed irrelevant studies through the selection process. In no step has AI been used to select or summarise literature, or for analysing and writing text.

Our search resulted in 2184 published studies (Table S1). We excluded studies that were not primary research studies published in peer‐reviewed international scientific journals, like reviews, conference reports or proceedings, book chapters, fact sheets, or preprints (*n* = 80; Figure 1). Based on the title of the study, we further excluded all studies not considering wild fish, like abiotic environmental parameters, other taxonomic groups, fisheries and aquaculture/stocking, fish as human food, toxins in fish and technical or methodological advances (*n* = 1110; Figure 1). For the remaining 994 studies, we screened the abstract, and in cases of uncertainty also the method section. We included empirical studies that addressed spatial or temporal variation of the pre‐defined outcomes (see Section 2.3) within the Baltic Sea, but excluded: (1) studies with the Baltic Sea as a single observation in comparison with other areas; (2) studies of fish in response to only fish management, eutrophication, physical exploitation, diseases, parasites and predation and toxins; and (3) pure modelling studies. It should be noted we included studies with output data from fish stock assessment models as projections of observational data, although these strictly are modelled data.

For the 297 studies remaining after reviewing the abstract for these criteria (Table S2), we screened the full text and classified the outcomes of the studies to hierarchical level and fish community (see Section 2.3). Species of marine origin that move between offshore areas for feeding and coastal areas for spawning, we here classify as demersal/pelagic species. Also, coastal species may use freshwaters for spawning, and we consider facultative anadromous species as coastal species. However, whitefish (*Coregonus maraena* Bloch 1779) occur in distinct sea‐spawning and river‐spawning populations (Säisa et al., 2008), and we therefore made a division between coastal and migrating ecotypes. We also considered vendace (*Coregonus albula* L. 1758) as a coastal species based on its freshwater origin despite foraging in pelagic habitats. Non‐indigenous species refer to species that are not native to the Baltic Sea and include both species that have been introduced through human activities and species that actively move into the Baltic Sea from their native range due to climate change.

For each study, we assessed if primary data were based on (i) field observation only or included experimental studies, (ii) in which time period (1975–1979, 1980–1984, …, 2015–2019, 2020–2025) a study had used data from, and for longer time periods the final year was always used, (iii) which ICES subdivisions (22–32; Figure S1) a study considered (for studies concerning or comparing several subdivisions, all subdivisions were counted), and (iv) recorded which traits were considered: physiological traits (metabolism, respiration, cardiac‐enzyme activity, etc.), reproductive traits (fertility, sperm performance, egg‐larvae hatching/survival), life‐history traits (somatic growth, mortality, fecundity), abundance (including spatial and temporal distributions) and community features (composition, diversity, predation rates). When possible, we scored if the relationship with each trait was significantly associated (either as *p* < 0.05 or ΔAIC < 0) to a climate variable, and if so the shape of the relationship (positive, negative, hump‐shaped or U‐shaped). Finally, (v) we noted if studies had assessed adaptive plastic‐ or evolutionary responses to change in climate variables. Studies were considered to assess adaptive plastic responses when comparing a trait or performance across an environmental gradient (reaction norm) of individuals from the same population. This included also field observations where individuals could be assessed under different conditions (e.g., back‐calculated growth from bone structures), but we excluded studies of short‐term responses along an environmental gradient (these could be considered as plastic responses but not necessarily adaptive). Studies were considered to assess evolutionary responses when comparing responses of individuals from different populations with contrasting environments (in space or time within the Baltic Sea) to similar environmental treatments (intra‐population variability was not considered enough to indicate evolutionary responses).

### 2.5 Ethical statement

This review only uses data of already published studies, which according to Estonian, Finnish and Swedish national laws does not require specific ethical permits. The care and use of experimental animals complied is therefore according to the specific permits of each published study, complying to respective countries' national animal welfare laws, guidelines and policies.

## 3 RESULTS

Water temperature (189 studies) and salinity (144 studies) were the most studied climate variables on fish in the Baltic Sea (Figure 2a), followed by studies considering oxygen and stratification (59 studies). There were 17 or fewer studies each considering ice, acidification, run‐off, and waves (Figure 2a). Studies on pelagic (104 studies) and demersal (98 studies) fish were most common, followed by coastal fish (85 studies). Only 19 and 15 studies focused on anadromous fish (no catadromous, like eel) and non‐indigenous species, respectively (Figure 2a). A majority, 175 studies, considered performance at the individual fish level, 105 studies population level performance, and 35 studied community level changes (Figure 2b). Only observational field studies dominated with 212 studies, and 85 studies included at least some controlled experiments. At the individual level, studies on reproductive and life‐history traits were most common, and at the population level abundance and recruitment (Figure 2c). Out of the 297 studies, 31 assessed some mode of adaptations, of which 17 assessed adaptive plastic and evolutionary adaptations in conjunction, 12 only plastic adaptive responses, and two studied only evolutionary adaptations (Figure 2d).

**FIGURE 2:**
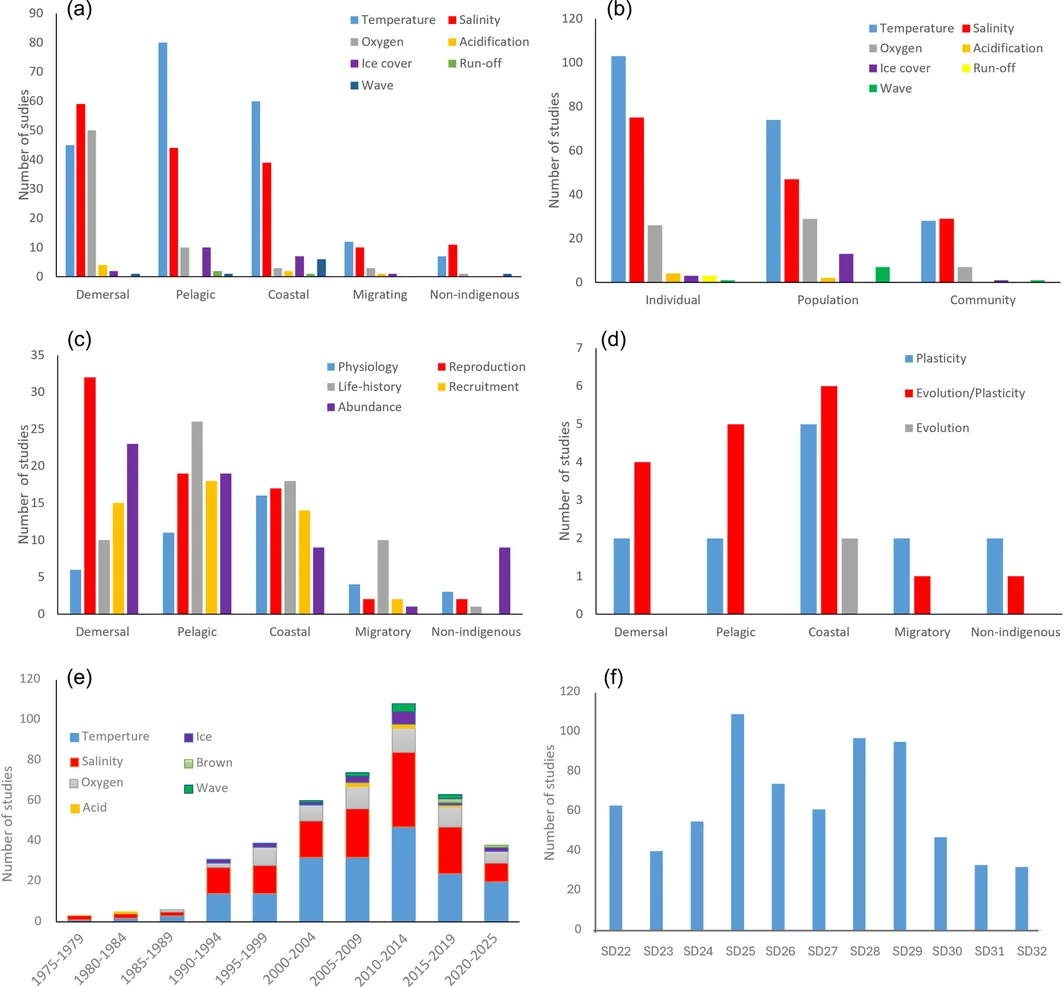
Number of studies included in in this review divided by (a) different climate variables per fish assembly, (b) different climate variables per hierarchical level, (c) different individual traits (physiology, reproduction, life‐history) and population performance (recruitment and abundance) divided by fish assembly, (d) mode of adaptation to climate variables divided by fish assembly, (e) study period (final year of data used, not publication year) and (f) ICES subdivision.

Starting from around 10 studies per 5‐year period during the 1970–1980's, there was an increase in the number of studies concerning climate variables to the peak of 75 studies in the period 2010–2014. Thereafter there has been a clear reduction in conducted studies (Figure 2e). However, there are reasons to believe there is a lag effect before studies in the latest period will be published but indicates a drop in the number of studies published. Responses to temperature and salinity dominated studies until 2020, when there was a reduction in studies concerning salinity. There were around 10 studies on responses of oxygen levels and stratification for each 5‐year period between 1990 and 2020 (Figure 2e). Most frequent were studies along an S‐NE axis in the Baltic Sea (ICES subdivisions 25, 28, 29) (95–109 studies per subdivision). The other subdivisions in the southernmost Baltic Sea (22, 24, 26) and central Baltic Sea (subdivision 27) had intermediate the number of studies (55–74 per subdivision), whereas northern and eastern subdivisions (30–32) and the Sound (subdivision 23) had the fewest (30–47 studies per subdivision) (Figure 2f).

### 3.1 Demersal fish

Temperature has a variable or weak effect on the performance of demersal fish (Figure 3a). Instead, increasing water temperatures in combination with high nutrient levels have contributed to less oxygen in demersal habitats (Saraiva et al., 2019), which have almost consistently resulted in a negative impact on several individual fitness and population performance parameters of demersal fish (Figure 3b). A large majority of studies show a positive relationship between salinity and individual level and population performance of demersal fish species (Figure 3c). Only a few studies considered acidification or ice dynamics on demersal fish (Figure 2a).

**FIGURE 3:**
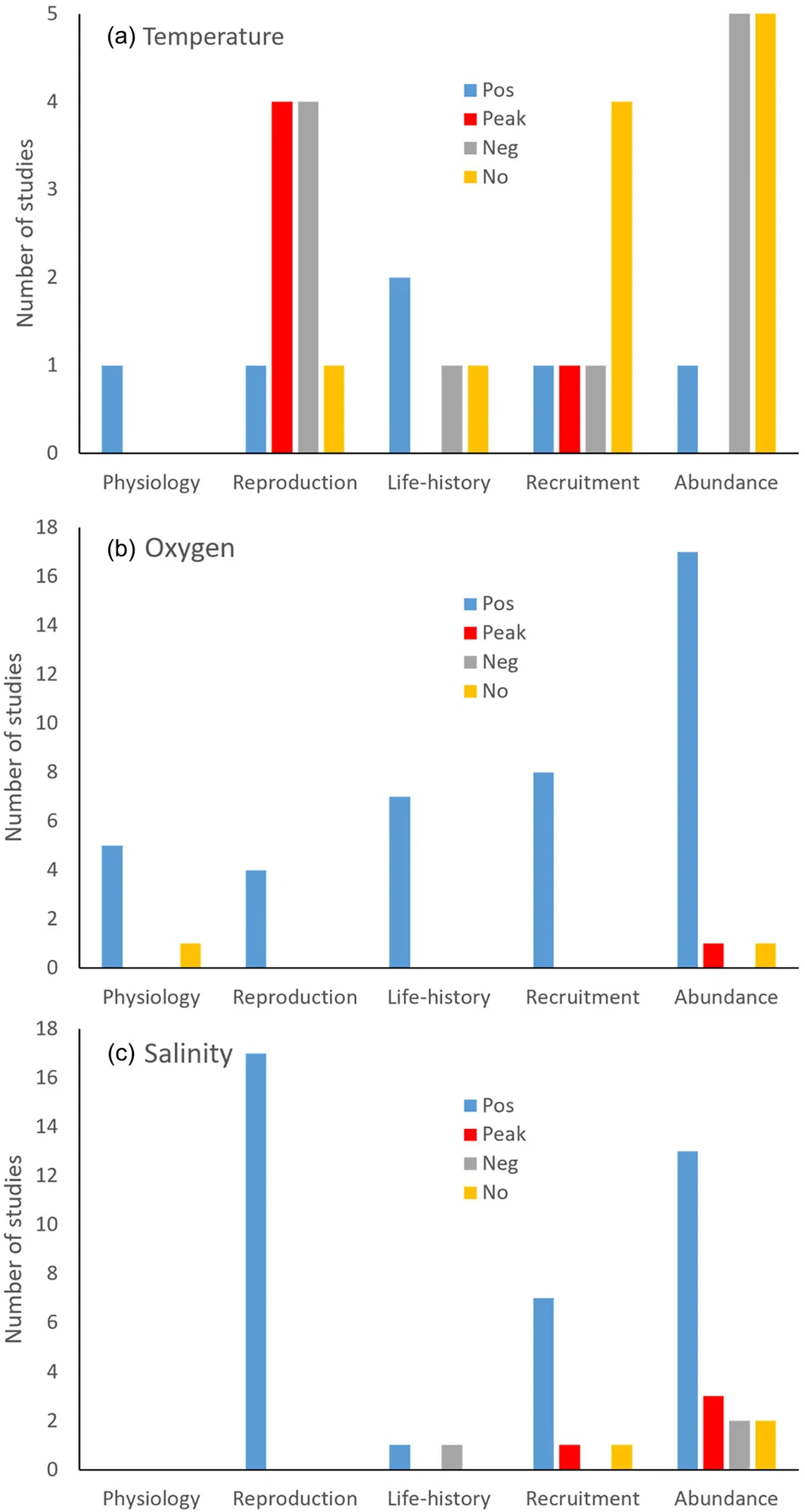
Number of studies on demersal fish showing positive (Pos), hump‐shaped (Peak), negative (Neg), or no (No) association with (a) temperature, (b) oxygen and (c) salinity divided per trait.

#### 3.1.1 Individual performance

Several experimental studies show that cod hatching success and larvae survival decrease above 8°C (Dahlke et al., 2016; Nissling, 2004; Politis et al., 2014), and peak around 12–17°C for turbot (*Scophthalmus maximus* L. 1958) (Kuhlmann & Quantz, 1980; Karas & Klingsheim, 1997; Nissling et al., 2006). Field surveys indicate that the peak in spawning time is delayed after warmer winter temperatures for cod (Oeberst & Bleil, 2003; Wieland et al., 2000), which may reduce the “window‐of‐opportunity” for the onset of independent feeding of cod larvae, with 1–3 days (Voss et al., 2012). On the other hand, field studies suggest individual condition, fecundity and body growth of cod (Heimbrand et al., 2024; Kraus et al., 2000; Lindmark, Anderson, et al., 2023) and body growth of European flounder (*Platichthys flesus*) to be positively correlated with water temperature (Smolinski & Mirny, 2017).

Experimental studies indicate that the minimum salinity levels required for successful reproduction of cod and flatfish are around 10–15‰ (Nissling et al., 2002; Petereit et al., 2014; Wieland & Jarre, 1997). However, the Baltic flounder (*Platichthys solemdali*) has adapted to lower salinity and can reproduce at 7‰ (Nissling & Larsson, 2018; Nissling & Wallin, 2020), and cod in the Ålands Sea can reproduce from 9‰ (Bergström et al., 2025). Thus, salinity levels already constrain reproduction and recruitment of most demersal fish species in a large part of the Baltic Sea, which could be further reduced if salinity levels decrease.

Only few studies address ocean acidification impacts on demersal fish in the Baltic Sea (Figure 2a), and the impact of pH on reproduction is variable among the few studies. For cod from the Bornholm basin (SD25), experimentally increased CO_2_ levels up to ~1400 μatm partial pressure in water (pCO_2_) had no impact on sperm motility relative ambient pCO_2_ (Frommel et al., 2010). Neither hatching nor survival of cod eggs form the Bornholm basin differed among experimentally increased pCO_2_ levels up to 3200 μatm (Frommel et al., 2013). In contrast, an experimentally increase from ambient to ~1000 μatm pCO_2_ on cod from the western Baltic (SD23) doubled larvae mortality rate (Stiasny et al., 2016).

Although many studies compare individual performance in relation to climate variables, we only found six studies investigating adaptations to climate variables. Four of these show that although reproductive performance of demersal fish increases with salinity (Figure 3c), there is evidence of evolutionary adaptations to lower salinity in cod (Bergström et al., 2025; Nissling & Westin, 1997) and flatfishes (Nissling et al., 2002; Nissling & Larsson, 2018; Nissling & Wallin, 2020). Hypoxia also shows consistent negative associations with individual performance of demersal fish (Figure 3b), but there is evidence that cod is capable of foraging within safe oxygen limits to avoid oxygen debts, indicating adaptive plastic response (in digestion rates) to hypoxia to avoid oxygen debts (Behrens et al., 2012), allowing them to forage in hypoxic habitats and migrate to oxygen‐saturated habitats for digestion (Neuenfeldt et al., 2009).

#### 3.1.2 Population performance

The associations between temperature and recruitment or abundance of demersal fish in the Baltic Sea differ between studies and species, with around half of studies showing no significant association (Figure 3a). In contrast, there are consistent positive associations between salinity or oxygen levels and recruitment or abundance (Figure 3b,c), where the exception is the European and Baltic flounder (MacNeil et al., 2025; Orio et al., 2017; Rau et al., 2019).

#### 3.1.3 Community changes

At the community level, there has been an increase in the relative abundance of temperate flatfish species (sole, turbot, dab) compared to boreal species (plaice) (Rau et al., 2019; Sparrevohn et al., 2013). Salinity is the main factor controlling species‐ and trait diversity in the southern Baltic Sea, followed by temperature and oxygen (Pecuchet et al., 2016, 2020; Smoliński & Radtke, 2017). Species richness (both total‐ and per sample diversity) increased in the Baltic Sea during the period 2000–2010, related to both increased temperature and salinity during this period (Hiddink & Coleby, 2012).

In addition to the direct effect of climate on demersal fish in the Baltic Sea, several studies show important food web effects from climate change. Contracted nursery grounds through reduced salinity and oxygen levels, for example, increase predation on egg and larvae of demersal fish (e.g., Köster et al., 2005; Neuenfeldt & Beyer, 2003; Orio et al., 2019; Sparholt, 1996; Voss et al., 2011). Condition and somatic growth of cod have also been shown to be negatively related to the abundance and intake of prey linked to climate variables (e.g., Casini et al., 2016; Lindmark, Anderson, et al., 2023; Neuenfeldt, 2002; Neuenfeldt et al., 2020; Spich & Fey, 2023).

### 3.2 Pelagic fish

Both experimental and observational studies show variable relationships between temperature and individual‐ and population performance parameters of pelagic fish but positive or hump‐shaped responses dominates (Figure 4a). Most studies indicate an overall positive, or weak, relationship between salinity and performance of pelagic fish (Figure 4b). Only 10 studies concern hypoxia (Figure 2a) showing no evident relationship to performance of pelagic fish in the Baltic Sea, and no studied concerned acidification. Instead, 11 studies addressed the impact of changes in ice coverage, showing evident impact on the phenology of pelagic fish but the performance differed between species and areas (e.g., MacKenzie & Köster, 2004; Polte et al., 2021). Four studies considered terrestrial run‐off, also showing variable responses.

**FIGURE 4:**
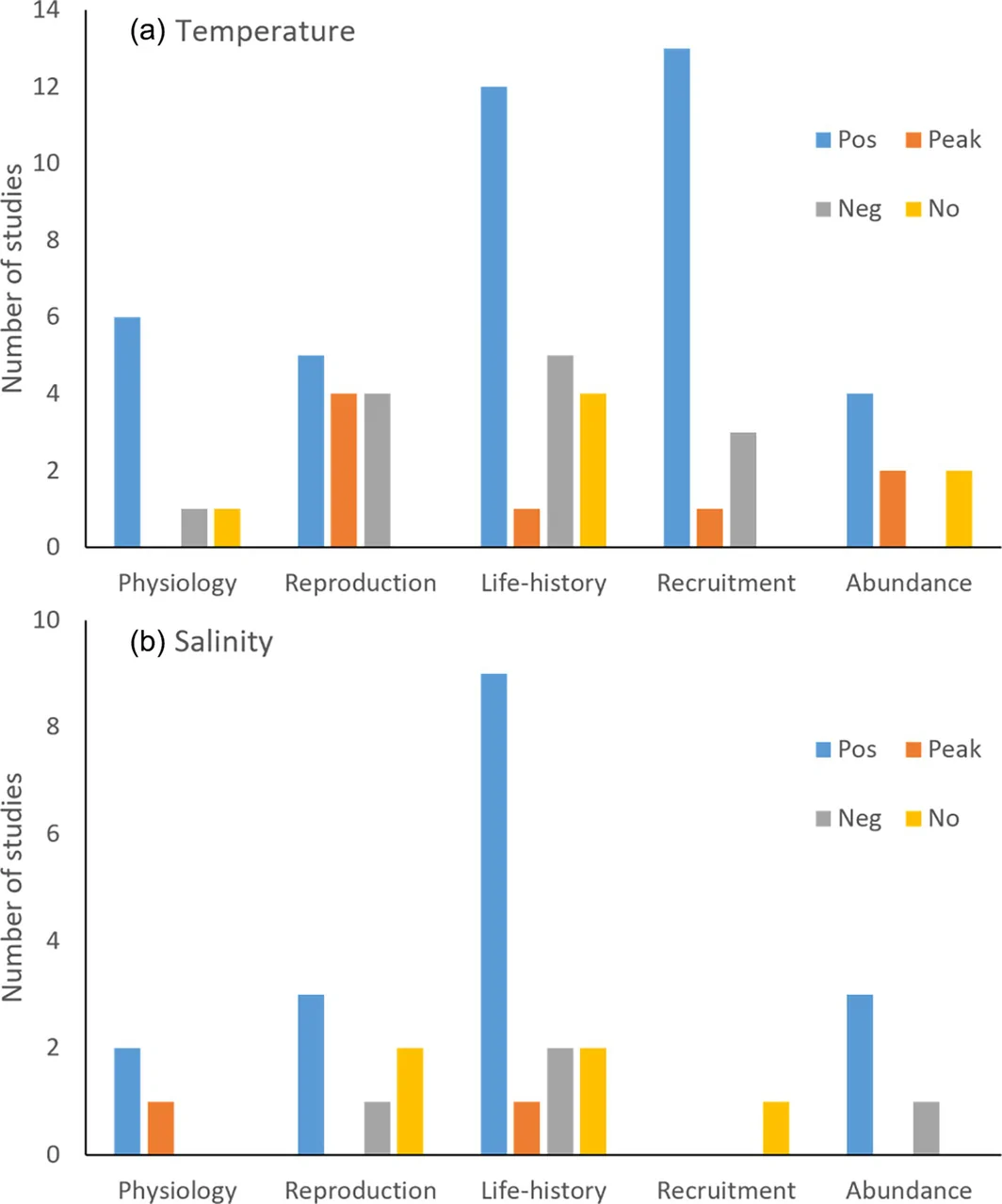
Number of studies on pelagic fish showing positive (Pos), hump‐shaped (Peak), negative (Neg), or no (No) association with (a) temperature and (b) salinity divided per trait.

#### 3.2.1 Individual performance

Many experimental and field studies indicate pelagic fish may in general perform better with increasing temperature (Figure 4a), but the between year variation may be low relative to seasonal changes (Huang et al., 2022, 2025). Above 15°C, herring shows an increased risk of lowered hatching rate (Peck et al., 2012), abnormal ovarian development (Ojaveer et al., 2015), smaller and more frequently malformed larvae (Mäkinen et al., 2023), and reduced larval growth due to increased metabolism (Arula et al., 2015; Fey, 2001; Huang et al., 2025). Experiments with sprat show peak larvae survival at 6–10°C (Petereit et al., 2008).

Individual herring performance peak between 8 and 16‰ (e.g. Berg et al., 2020; Lahti, 1989; Poirier et al., 2017; Rajasilta et al., 2021) and several studies show salinity is positively associated with somatic growth (Casini et al., 2006; Flinkman et al., 1998; Rajasilta et al., 2011; Rönkkönen et al., 2004). For sprat, salinity is, weakly, positively associated with body condition (Cardinale et al., 2002; Casini et al., 2006) and fecundity (Haslob et al., 2011), but egg mortality was higher in a year following a major saltwater inflow (Voss et al., 2011). Also, somatic growth of sprat, as indicated by otolith increment, decreases with salinity (Smoliński & Gutkowska, 2024). Both sprat and herring do diel migrations across the halocline, but sprat is less restricted relative to oxygen levels compared to herring (Neuenfeldt, 2002; Neuenfeldt & Beyer, 2003).

Besides temperature and salinity, Smolinski (2019) found a positive association between terrestrial run‐off and body growth of herring in the southern Baltic Sea, likely as it stimulates prey productivity. In contrast, Dippner et al. (2019) suggested terrestrial run‐off will lower herring mean body mass through lower prey quality. Increased browning from precipitation tends to lower juvenile somatic growth of three‐spined sticklebacks (Bell et al., 2022).

Five studies have assessed adaptations to variation in temperature and three studies to variation in salinity in pelagic fish. Baumann et al. (2006) showed that sprat larvae from northeastern areas (and born later in the year) had higher somatic growth rates at a given temperature than larvae from the western part of the Baltic Sea, indicating a local temperature regime adaptation. Similar, Oeberst et al. (2009) suggested herring larvae in the southern Baltic Sea to have steeper reaction norms in growth to temperatures than in more northern areas as an evolutionary adaptation to warmer waters and higher prey production in southern areas. In contrast, Petereit et al. (2008) could not find any difference in egg development rate in relation to temperature among sprat from different parts of the Baltic Sea, indicating no local evolutionary adaptation to temperature for this trait. Both Schaarschmidt et al. (1999) and Lefébure et al. (2011) show acclimatation to temperature in adult three‐spined sticklebacks, and sticklebacks sampled in fall/winter also had lower growth rates at identical temperatures than fish sampled in spring (Lefébure et al., 2011), indicating plastic adaptation to avoid starvation if water temperature increases in winter.

In a common garden experiment, Poirier et al. (2017) found that fertilisation rate did not differ with salinity 7–20‰ for herring from 20‰, whereas herring from 7‰ had higher fertilisation at 7‰ but reduced fertilisation at higher salinity. Hence, herring from low salinity seems to have evolutionary adapted to lower salinity conditions by trading off with lower reproduction in high salinity. For egg hatching rate there was no differences between populations or environments (Poirier et al., 2017). Using common garden experiments, Schaarschmidt et al. (1999) and Heckwolf et al. (2018) showed three‐spined stickleback from different salinity can adapt to high salinity, survival is only reduced at true marine conditions (>30‰) for individuals from the least saline environments, whereas all populations survive brackish (6‰) conditions (Heckwolf et al., 2018). However, Schaarschmidt et al. (1999) showed that even when acclimated to freshwater (0.3‰) there is an increased mortality among three‐spined stickleback from the Baltic Sea indicating there is a lower salinity level they can adapt to.

#### 3.2.2 Population performance

Warmer waters and shorter periods with ice cover are associated with earlier and increased recruitment of sprat (Baumann et al., 2008; MacKenzie & Köster, 2004; Margonski et al., 2011). According to Haslob et al. (2012), sprat population growth should peak at a water temperature around 4°C above the mean ambient temperature 1976–2008. The relationship between recruitment and temperature differs between herring stocks in the Baltic Sea. For the central and northern stocks, recruitment seems to be favoured by warmer summer temperatures or milder winters (Bartolino et al., 2014; Margonski et al., 2011; Ojaveer et al., 2021). In contrast, higher temperatures early in the year during larval development can explain lower recruitment of south‐western (autumn‐spawning) Baltic herring and a mismatch in peak larval abundance with optimal temperature windows for feeding (Dodson et al., 2019; Livdāne et al., 2025; Moyano et al., 2020; Polte et al., 2021).

#### 3.2.3 Community changes

Although hypoxia seems to exert little direct impact on pelagic fish, the reduction in cod abundance due to hypoxia has resulted in a shift in the Baltic Sea towards a pelagic fish‐dominated food web from the late 1980s (Casini et al., 2008; Möllmann et al., 2009; Österblom et al., 2007). In addition, the total biomass of pelagic fish is positively associated with water temperature (Alheit et al., 2005; Orlowski, 2003), while species composition is mainly determined by salinity (Karaseva et al., 2012; Pekcan‐Hekim et al., 2016).

Several of the observed patterns of pelagic fish with temperature and salinity are as for demersal fish partly mediated through changes in prey abundance and composition (Arula et al., 2015; Hinrichsen et al., 2010; Möllmann et al., 2005; Ojaveer et al., 2021). Zooplankton abundance and composition, related to hydroclimate variation, correlate with herring body growth and abundance (Dippner et al., 2019; Livdāne et al., 2025; Möllmann et al., 2005; Rajasilta et al., 2019). Interspecific competition over zooplankton between sprat and herring has reduced herring body growth (Casini et al., 2010; Smolinski, 2019; Smoliński & Haase, 2025) as sprat are better competitors for small‐sized (<20 μg) zooplankton (Ojaveer et al., 2018) that are characteristic of lower salinity conditions (Flinkman et al., 1998).

### 3.3 Coastal fish

Coastal fish in the Baltic are phylogenetically diverse, harbouring both freshwater and marine fish; hence, exhibiting diverse responses to climate change. Studies on species of a freshwater origin dominated the literature and thereby positive associations to temperature (Figure 5a) and negative responses to salinity (Figure 5b) dominated, but there was evident study‐to‐study variation (Figure 5).

**FIGURE 5:**
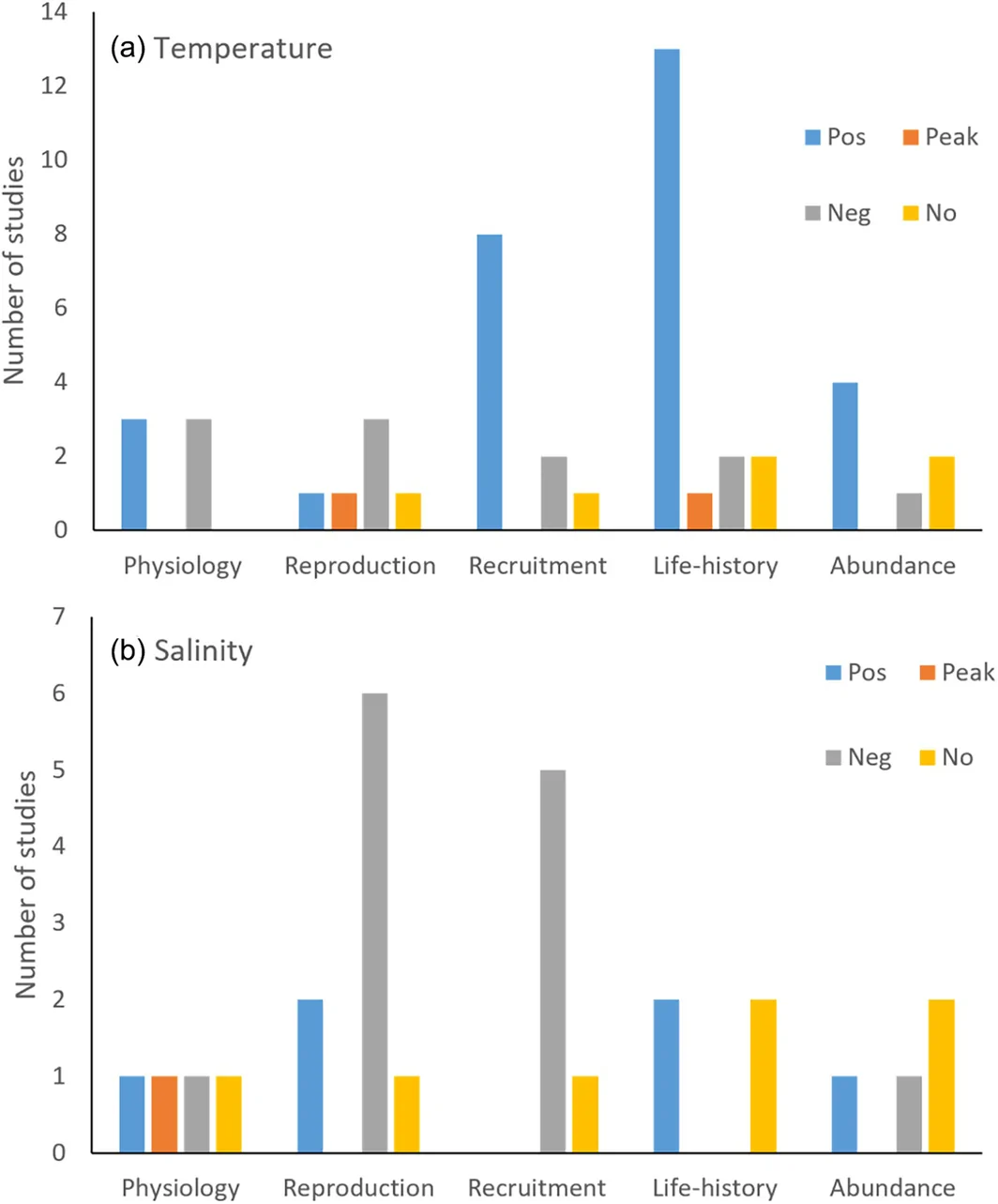
Number of studies on coastal fish showing positive (Pos), hump‐shaped (Peak), negative (Neg), or no (No) association with (a) temperature and (b) salinity divided per trait.

#### 3.3.1 Individual performance

Both observational and experimental studies of coastal fish of a freshwater origin show that although egg quality and hatching success decrease with temperature (Bruneaux et al., 2014; Luksiene & Sandström, 1994; Niu et al., 2023; Sandström et al., 1995, 1997), larval survival and body growth increases with water temperature (Figure 5a). The positive impact of temperature diminishes as some larger fish species, such as perch (*Perca fluviatilis* L. 1758), pike (*Esox lucius* L. 1758) and pikeperch (*Sander lucioperca* L. 1758), get older or even become negative for larger individuals (Berggren et al., 2022; Huss et al., 2019; Lindmark, Karlsson, & Gårdmark, 2023; van Dorst et al., 2023). This can partly be due to reduced size at maturation with increased temperature (Pilipaitytė et al., 2025). Adult perch also face elevated mortality at high temperatures (Lindmark, Karlsson, & Gårdmark, 2023; Sandström et al., 1995), but this is not evident among adult pike populations (Berggren et al., 2022).

Coastal fish of freshwater origin show negative responses to salinity (Figure 5b), however, freshwater species like pike, perch, roach (*Rutilus rutilus* L. 1758), ruffe (*Gymnocephalus cernua* L. 1758) and sea‐spawning whitefish can spawn in salinities up to ~10 ‰ (Christensen et al., 2016; Jørgensen et al., 2010; Papakostas et al., 2012; Skovrind et al., 2013; Tibblin et al., 2012; Vetemaa & Saat, 1996). Egg hatching and larval survival can, on the other hand, be reduced at already 4–5‰ for some freshwater species (Bohlen, 1999; Schofer, 1979).

Perch may experience decreased body growth in darker waters (Huss et al., 2021; van Dorst et al., 2023), which is expected to become darker due to increased terrestrial run‐off of humic substances. Shorter ice duration seems to enhance winter survival of young‐of‐the‐year pikeperch (Lappalainen, Erm, et al., 2000) and gobies (Parmanne & Lindström, 2003). There are few studies concerning the impact of acidity on coastal fish. Both Urho et al. (1990) and Alter and Peck (2021) suggested that low pH may reduce the reproduction of coastal spawning fish in the Baltic Sea.

There was in total 14 studies investigating adaptive plastic and evolutionary adaptations in coastal fish, of both freshwater and marine origin, so results are variable. Physiological responses to temperature variation in perch indicate rapid adaptive plastic responses to temperature, whereas longer‐term acclimation shows counter‐gradient variation (Sandblom, Ekström, et al., 2016; Sandblom, Clark, et al., 2016). However, both perch acclimated to or with an evolutionary history in warmer temperatures seem to lose plastic cardiac responses to temperature variation (Pichaud et al., 2019; Sandblom, Clark, et al., 2016). One reason for this may be that too high cardiac capacity induces oxidative stress (Pichaud et al., 2020). Ekström et al. (2021) further suggested that warm adapted perch also have higher tolerance to hypoxia, further indicating individual evolutionary adaptations to climate change. A longitudinal study of perch body growth shows small differences in reaction norms across a temperature gradient, indicating weak local adaptations or stabilising selection (Lindmark et al., 2025). Taken together, these studies indicate Baltic Sea perch can adapt to increasing temperature both on short‐ and long‐term time perspectives.

Coastal species of a marine origin instead show poor ability for adaptations to warmer water. Eelpout (*Zoarces viviparus*) cannot acclimate to temperatures above 18°C (Zakhartsev et al., 2003), and individuals acclimatised above 12°C have increased oxygen consumption much faster with (acute) increasing temperature than eelpouts acclimated at 3°C. Respiration and swimming activity also decline with acute decreasing oxygen levels for eelpout, although they can survive hypoxic conditions (Fischer et al., 1992). While also the lesser sand eel (*Ammodytes tobianus*) shows increased metabolic rates with acute temperature increase, it does not show any behavioural response or changes in swimming speed to avoid hypoxia following the temperature increase (Behrens & Steffensen, 2007).

Several coastal species of marine origin also show adaptive plastic responses to salinity. Acute (3 days) changes in salinity (both up and down) activated the innate immune system of broad‐nosed pipefish (*Syngnathus typhle*) but reduced the specific immune response (Birrer et al., 2012). Pipefish (*S. typhle*) from higher salinity have steeper reaction norms in relation to salinity than individuals from lower salinity (Goehlich et al., 2021), indicating more constitutive responses to salinity for pipefish from lower salinity. Eggs from sand goby (*Pomatoschistus minutus*) had increased hatching success and reduced egg infections as salinity increases from 6 to 16 ‰ (Lehtonen & Kvarnemo, 2015), but Lindström et al. (2021) suggested that sperm performance in three species of gobies was higher at brackish condition (6‰) than in marine conditions (31‰). Acclimatation to different salinities affected nesting behaviour of sand gobies from a low salinity population (Lehtonen et al., 2016), and Mück and Heubel (2018) also found different nesting behaviour of sand gobies along a salinity gradient in the Baltic Sea.

#### 3.3.2 Population performance

Although strong effects on individual performance, the influence of temperature on abundances of fish of freshwater origin are low or uncertain (Bergström et al., 2016; Lehtonen et al., 2009; Östman, Lingman, et al., 2017), but in the northern Bothnian Bay, an increase in pikeperch abundance is likely an effect of warmer waters (Olin et al., 2023). Also, variation in salinity levels <6‰ seems to have little impact on abundance and distribution of coastal fish populations of freshwater origin (Dainys et al., 2019; Östman, Lingman, et al., 2017). The salmonid vendace (*Coregonus albula* L.1758), in contrast, have lower recruitment in years with high winter temperature or salinity (Bergenius et al., 2013) and the spatial distribution is compressed at warm winter temperatures and less ice cover (Veneranta et al., 2013).

#### 3.3.3 Community changes

Coastal fish communities are species rich relative to other fish communities in the Baltic Sea, and changes in composition are mainly attributed to changes in salinity, with marginal effects of temperature (Olsson et al., 2012; Smoliński & Całkiewicz, 2015; Törnroos et al., 2019). Species richness generally increases with salinity (Koehler et al., 2021; Lappalainen, Shurukhin, et al., 2000), whereas mean trophic level increases with water temperature (Östman, Lingman, et al., 2017). In an experiment, Svensson et al. (2017) found that top‐down control by perch was stronger in an artificially heated area, which indicates a faster flow of resources to higher trophic levels with increasing temperature. Total coastal fish abundance is positively associated with water temperature in the central Baltic Sea (Snickars et al., 2009; Thorman & Wiederholm, 1984), but in the Åland Islands, increased temperature is associated with a decline of benthic‐feeding fish (Snickars et al., 2015).

### 3.4 Migrating fish species

Although salmonids are adapted to cooler environments, body growth of (wild) Atlantic salmon (*Salmo salar* L. 1758), brown trout (*S. trutta* L. 1758) and whitefish all increase with temperature (Friedland et al., 2017; Kallio‐Nyberg et al., 2004, 2019; Lejk et al., 2021). For whitefish, higher water temperature is associated with earlier maturation (Veneranta et al., 2021). Peak return day to spawning rivers is earlier with increasing sea temperatures for salmon but not for trout (Dahl et al., 2004). For salmon, an increased temperature seems to come with increased oxidative stress (Kanerva et al., 2020) and reduced smolt survival (Friedland et al., 2017; Jutila et al., 2005). Temperatures below 9°C may, however, also reduce smolt survival (Jutila et al., 2005).

Friedland et al. (2017) found a general positive association between post‐smolt survival and oxygen levels in salmon, whereas survival was negatively associated with salinity in the southern Baltic Sea and positively correlated in the northern Bothnian Bay. Vuori et al. (2008) suggest that lower salinity may be associated with reduced food quality for salmon, resulting in oxidative stress and increased fry mortality (M74 syndrome). Egg fertilisation in sea trout is possible up to 6‰ but hatching duration increases with salinity, and no hatching is observed above 4‰ (Landergren & Vallin, 1998; Rubin, 1994).

At the population level, Huusko and Hyvärinen (2012) suggested using a 200‐year time series, a trade‐off between larger salmons but lower abundances during warmer periods with high riverine run‐off and the opposite during colder and drier periods, indicating climate‐driven density dependence.

### 3.5 Non‐indigenous species

Of the 15 studies concerning invasive or fugitive species in the Baltic Sea in relation to climate variables, around half consider the invasive round goby (*Neogobius melanostomus* Pallas 1814). Experimental studies show that round gobies can acclimatise well up to at least 28°C and tolerating water temperatures well above 30°C, but their temperature preference does not change with acclimatisation temperature (Christensen et al., 2021). Field observations over seasons indicates round gobies mainly occupy the warmest depth strata, likely an effect of the Baltic Sea generally being cooler than their preferred 18–24°C (Behrens et al., 2022). Hence, increasing water temperatures should increase the amount of suitable habitat for round goby (Quattrocchi et al., 2023), but a field study indicates a weak relation between abundance and temperature (Lewin et al., 2023).

Several experimental studies on round goby show that the low salinity in parts of the Baltic Sea increases physiological stress (Puntila‐Dodd et al., 2021), but also that stress increases and body growth decreases above 15‰ (Hempel & Thiel, 2015; Behrens et al., 2017). Salinity also has a strong impact on reproduction. Sperm performance of round goby from the Baltic Sea shows no acclimation to freshwater (Green et al., 2021), but although sperm performance peaks at 10–15‰, there are ongoing adaptations to both lower and higher salinity conditions at the invasion fronts indicating evolutionary adaptations (Green et al., 2020). Along the German Baltic Sea coast, low salinity (<15‰) is the main factor constraining round goby abundance (Lewin et al., 2023).

Increasing water temperatures, in combination with saltwater inflows, would facilitate the expansion and survival of new species from the Atlantic into the Baltic Sea (Hinrichsen et al., 2022; Montero‐Serra et al., 2015; Więcaszek et al., 2023), but current salinity levels and temperatures during winter are still below the tolerance limits for these species (Hinrichsen et al., 2022). Climate change could potentially increase the risk for new invasions of human related introductions of species from other brackish seas, for example, tubenose goby (*Proterorhinus semipellucidus* Kessler 1877) (Kurina et al., 2024; Truuverk et al., 2021), but so far there are few documented examples in the scientific literature.

## 4 DISCUSSION

This review includes a broad range of species and habitats showing variable associations to climate variables, but we find the strongest empirical support from both experimental and observational studies for: (i) reduced available habitat for reproduction and foraging of marine demersal fish species due to oxygen limitation and low salinity, resulting in poorer condition, growth and reproduction, and (ii) an increased recruitment and body growth due to warmer waters of juveniles and small adults of coastal fish species of freshwater origin and some small pelagic fish. Several studies further demonstrate that fish species in the Baltic Seas show adaptive plastic or evolutionary responses to climate change, suggesting fish species, at least partly, adapt to the changing conditions.

### 4.1 Climate change impacts

The Baltic Sea is globally one of the seas most affected by climate change (Belkin, 2009), and future climate trends will likely continue to impact the abiotic conditions in the Baltic Sea (Dutheil et al., 2022). An increase in water temperature is an evident abiotic effect of climate change in the Baltic Sea, but responses are variable among species (Figures 3, 4, 5) or even within species, for example, herring (Moyano et al., 2020; Oeberst et al., 2009; Ojaveer et al., 2021). Moreover, despite evident temperature effects in many studies on individual traits such as metabolism, food intake rate, growth, reproduction rate, survival and phenology across taxa, considerably fewer studies report changes in population abundance, size structure and community composition related to water temperature (Figures 3, 4, 5).

Increased temperature in combination with the historic high nutrient loadings and changed stratification has resulted in large hypoxic areas, and these are predicted to increase further unless a reduction in nutrient loadings to the Baltic Sea (Saraiva et al., 2019). Hypoxia has caused a major contraction of available habitat space for reproduction and foraging of many marine demersal fish (Behrens et al., 2012; Eero et al., 2015, 2023; Limburg & Casini, 2018, 2019; Neuenfeldt, 2002; Neuenfeldt et al., 2009), and although indications of ongoing adaptations to lower oxygen levels (Behrens et al., 2012), hypoxia will likely be the major constraint for demersal fish in the future.

Salinity is an important abiotic constraint for all fish in the brackish Baltic Sea, like in other marginal seas and estuaries (Bat et al., 2024; Gillanders et al., 2011). There is a natural variability in salinity levels at a decadal scale in the Baltic Sea, and although uncertain, predicted to decline towards the end of this century (Saraiva et al., 2019). Most studies on demersal and pelagic fish of marine origin in the Baltic Sea show positive responses to increasing salinity on reproduction, condition and abundance, which with some exceptions typically cannot reproduce below 9‰ (Bergström et al., 2025; Nissling et al., 2002; Rajasilta et al., 2011; Voss et al., 2011). On the other hand, salinity levels higher than 5–10‰ tend to constrain reproduction of fish of freshwater origin (e.g., Skovrind et al., 2013; Tibblin et al., 2012; Vetemaa & Saat, 1996), and above 15‰ for the invasive round goby (Green et al., 2020, 2021; Lewin et al., 2023). Salinity was also the main factor explaining temporal variation in fish community composition and richness (Hiddink & Coleby, 2012; Olsson et al., 2012; Pecuchet et al., 2016, 2020; Rau et al., 2019). Hence, changes in salinity will likely impact Baltic Sea fish, but variation in responses between fish of different evolutionary origin and uncertainty in salinity trends make predictions elusive.

There were considerably fewer studies addressing the impact of other climate‐related variables. Ocean acidification is of concern at the global scale (reviewed by Doney et al., 2020), but despite a decrease in pH that can be expected with climate change in the Baltic Sea (Gustafsson & Gustafsson, 2020), only a few studies are available on the effects of ocean acidification on fish in the Baltic Sea, which show contradicting results (Alter & Peck, 2021; Frommel et al., 2010, 2013; Stiasny et al., 2016). The limited number of studies addressing impacts of ice cover, terrestrial run‐off and waves makes any general conclusions regarding the importance of these climate drivers uncertain.

### 4.2 Adaptations to climate change

Several studies show adaptive plastic or evolutionary responses can reduce the impact of climate change at an individual level. For example, adaptive plastic and evolutionary responses can partly counteract the impact of hypoxia in cod (Behrens et al., 2012), heat tolerance in perch (Pichaud et al., 2019) and tolerance to salinity variation in pelagic fish (Heckwolf et al., 2018; Poirier et al., 2017) and round goby (Green et al., 2021). However, the adaptation to new climate conditions in many studies seems to come with a cost, either directly as increased physiological stress (Pichaud et al., 2020; Puntila‐Dodd et al., 2021) or elevated mortality, or indirectly by lowered performance in other environments (Green et al., 2021) or susceptibility to infections (Goehlich et al., 2021; Poirier et al., 2017). Although the number of studies is not large, some studies indicate that coastal fish species adapted to new climate environments seem to show more constitutive responses to environmental variation than under more native conditions (Goehlich et al., 2021; Pichaud et al., 2019; Sandblom, Clark, et al., 2016).

The empirical evidence for evolutionary and plastic adaptations suggests that reaction norms to climate variables may have already changed or is changing for fish species in Baltic Sea due to ongoing climate change. Despite that we did not here include pure modelling studies, we are not aware of any study on fish in the Baltic Sea considering eco‐evolutionary feedback mechanisms in relation to climate change, that is, how changes in reaction norms due to climate variation may modifies population level responses.

### 4.3 Differences across hierarchical levels

Variation in both temperature and salinity had a strong impact on several individual traits and performance, but this may not evidently translate into changes at a population or community level. For example, the positive effect of increased temperature on body growth, reproduction and recruitment for some coastal and pelagic species was not or only weakly influencing abundance or size distributions at the population level. There is empirical evidence that changed performance in one trait may be compensated by an opposite impact in another trait. For example, increased recruitment and body growth in smaller perch seem to be counteracted by increased mortality (Lindmark, Karlsson, & Gårdmark, 2023; Sandström et al., 1995), and density dependent processes in later life stages can counteract climate induced changes in earlier life stages (Arula et al., 2022; Casini et al., 2011, 2016).

Another reason for a weak link between individual and population levels may be the indirect effects of climate change. Especially for pelagic fish, the variation in temperature and salinity not only affects fish but also the phenology, composition and abundance of zooplankton that may be more important drivers of population dynamics and size distributions (Dippner et al., 2019; Möllmann et al., 2005; Rajasilta et al., 2019). The link between individual performance and population dynamics is also weakened by species are affected by other drivers and human pressures, most notably the fisheries for marine species (Casini et al., 2010; Froese et al., 2022; Österblom et al., 2007). For coastal fish, eutrophication, physical exploitation and apex marine predators can locally impact fish abundances and size structures (Blenckner et al., 2021; Berggren et al., 2022; Laur et al., 2014).

### 4.4 Appraisals and knowledge gaps

Species targeted by commercial and recreational fisheries dominate among the reviewed studies, with the addition of the invasive round goby. This is not surprising given their importance for humans. There are only sporadic studies on relatively abundant families such as sand‐eels (Ammodytidae), cyprinids (Cyprinidae), gobies (Gobiidae) and pipefish (Syngnathidae), indicating significant knowledge gaps at the fish community level. At the same time, the few studies available indicated similar responses as other fish species based on habitat use and evolutionary history (marine/freshwater).

Studies from the central parts of the Baltic Sea Proper (ICES subdivisions 25–29) were most frequent, whereas studies concerning Gulf of Bothnia (SD 30–31), relative to its size, were underrepresented. Reasons for this may include fewer universities in the area and fewer species, but also that species of marine origin may face both warmer and anoxic water in the south and desalination in the north. However, several of the studies on demersal and pelagic fish of marine origin (cod, herring and sprat) in the Western (SD 22–24) and Southern to Central Baltic Sea (SD 25–29) use data from the very same scientific fish surveys and stock models by ICES. Although studies may use different time periods, data selection, or analytical methods, they do not represent independent cases, resulting in inflated numbers of responses to climate variables for these populations. The number of studies showing specific associations to a climate variable should therefore not be interpreted as independent observations but as (somewhat) different evidence for the same populations.

As we here only consider qualitative associations, we have not estimated any effect size or in any other way considered within‐study variability, sample sizes or any other quality of the observed relationship to climate variables. Again, especially for the commercial stocks or for those species where population responses are based on trend analyses, they stand out as the sample size may effectively be one, with no control. However, these have been replicated across studies (see above). There is hence a need to address study quality and effect sizes for smaller subsets of studies in the future.

Care should always be taken when predicting future responses to climate change, as most of the studies infer patterns from past observational studies, often relying on time series or a space‐for‐time approach. It is always challenging to single out the importance of single variables when addressing naturally variable systems (Meier et al., 2022), especially for studies with low sample sizes, to predict future changes based on historic patterns. Fish in the Baltic Sea are further subject to multiple human pressures (Blenckner et al., 2021) that can cause cumulative or interactive responses, making them more sensitive than to a single specific climate variable. For some demersal species, the ‘reproductive volume’ has been used as an index to integrate several pressure variables on reproduction (MacKenzie et al., 2000; Nissling et al., 2002; Ustups et al., 2013), and the development of corresponding and integrated indices would be valuable for other species and life stages.

Experiments manipulating climate variables is also required to scrutinise responses to specific variables relevant to future climate changes in the Baltic Sea but were considerably fewer than observational studies for fish in the Baltic Sea. Experimental studies are due to practical constraints biased towards individual physiological and reproductive traits and performance at egg‐larval stages. Although there are examples of contrasting results between experimental and observational studies, experimental studies confirm the most evident observational patterns, for example, the negative impact of hypoxia and desalination on demersal fish, increased body growth and reproduction of small pelagic and coastal fish of freshwater origin.

A major gap for understanding and predicting future responses of fish to climate change in the Baltic Sea is how fast species can adapt to the new environmental conditions and its implication on population dynamics. Only a few studies consider evolutionary adaptations but show that fish are adapting to the new environmental conditions (Behrens et al., 2012; Green et al., 2020; Pichaud et al., 2019; Poirier et al., 2017). However, the consequences of these adaptations on survival and recruitment, and in the longer run population dynamics, are not well understood. On the other hand, it is reasonable to assume that such adaptations may have already been incorporated in the observed outcomes of studies considering long‐term (decadal or longer) population dynamics. To what degree evolutionary adaptations have counteracted, or reinforced, the effect of climate change in Baltic Sea fish is currently unknown.

### 4.5 Future perspectives

As future climate change may be accelerating in the Baltic Sea region (Dutheil et al., 2022) with more frequent extreme events (Rutgersson et al., 2022), it will be of essence to understand how changes in reaction norms will be translated into changes in population dynamics and demography, and in the end community dynamics, to predict and manage fish resources under different climate change scenarios. We want to stress that we here only considered climate change impacts on fish in isolation from other stressors, and there is a need to develop better understanding and predictions of the consequences of interactive effects of climate changes and other human pressures, for example, eutrophication, contaminants, habitat degradation and fishing mortality. To accommodate future climate change, a precautionary approach to management is therefore important to safeguard species adaptability to reduce deleterious impacts from multiple stressors from a likely even more pronounced changing climate in the coming decades.

## AUTHOR CONTRIBUTIONS

**Örjan Östman:** Conceptualisation (lead); data curation; formal analysis; investigation; methodology; visualisation (lead); writing—original draft; writing—review and editing. **Meri Kallasvuo:** Methodology; validation; writing—review and editing. **Sanna Kuningas:** Methodology; validation; writing—review and editing. **Antti Lappalainen:** Methodology; validation; writing—review and editing. **Noora Mustamäki:** Methodology; validation; writing—review and editing. **Rahmat Naddafi:** Methodology; validation; writing—review and editing. **Lauri Saks:** Methodology; validation; writing—review and editing. **Jens Olsson:** Conceptualisation (supporting); methodology; project administration; validation; visualisation (supporting); writing—review and editing.

## FUNDING INFORMATION

This work has been financed by the Swedish Agency for Marine and Water Management (projects 00735‐20 and 1449‐23) and the Strategic Research Council (grants 292985 and 314225).

## Supporting information


**Table S1.** All literature sources resulting from search at Web of Science. Publications was then included (1) or excluded (0) based on the selection criteria.

**Table S2.** Outcomes of published studies assessed in this review. For each study we have assessed plastic or evolutionary adaptations and which traits are considered and at what heirarchical level, if the study involves experiments (E) or only observational field studies (F). The table shows which climate variables each study consider and the shape of the response (‘+’ positive, ‘0’ no sign. response, ‘−’ negative, ‘U' shaped, ‘P' hump‐shaped).

**Figure S1.** Map over the Baltic Sea and sub‐basins and ICES subdivisons (SD). Grey lines and blue text show the boundaries between different basins in the Baltic Sea and Kattegat, whereas orange lines and text indicate ICES subdivisions. Illustration modified from HELCOM sub‐basins of the Baltic Sea https://gis.ices.dk/geonetwork/srv/api/records/225df9db-bfdf-4388-8ccb-fa4b99053a36/attachments/subbasins.png.

## Data Availability

A list of reviewed studies and information about each study is available as Figure S1, Tables S1 and S2.
