# Supplementary material for: Climate change impacts on fish in the Baltic Sea
Source: J Fish Biol. 2026 Jul 26;109(4):2147–65. doi: 10.1111/jfb.70582 (PMC13675489; doi:10.1111/jfb.70582)
Supplement: Supplementary file 1 [file JFB-109-2147-s001.docx]

Supplementary Figure S1: Map over the Baltic Sea and sub-basins and ICES subdivisons (SD). Grey lines and blue text show the boundaries between different basins in the Baltic Sea and Kattegat, whereas orange lines and text indicate ICES subdivisions. Illustration modified from HELCOM sub-basins of the Baltic Sea <https://gis.ices.dk/geonetwork/srv/api/records/225df9db-bfdf-4388-8ccb-fa4b99053a36/attachments/subbasins.png>.
